# An Antiracism Community-Based Participatory Research With Organizations Serving Immigrant and Marginalized Communities, Including Asian Americans and Native Hawaiians/Pacific Islanders in the United States Pacific Northwest: Qualitative Description Study With Key Informants

**DOI:** 10.2196/43150

**Published:** 2023-01-11

**Authors:** Connie Kim Yen Nguyen-Truong, Sara F Waters, Meenakshi Richardson, Natasha Barrow, Joseph Seia, Deborah U Eti, Keara Funchess Rodela

**Affiliations:** 1 College of Nursing in Vancouver Washington State University Vancouver, WA United States; 2 Human Development Department in Vancouver College of Agricultural, Human, and Natural Resource Sciences Washington State University Vancouver, WA United States; 3 Human Development Department in Vancouver Washington State University Vancouver, WA United States; 4 College of Nursing Health Sciences Spokane Washington State University Spokane, WA United States; 5 Lived Experience Coalition Federal Way, WA United States; 6 Community Health Worker Common Indicators Project Seattle, WA United States

**Keywords:** Asian Americans, Native Hawaiians/Pacific Islanders, community-based participatory research, qualitative description, coalition, antiracism, racial discrimination, race-based stress, racial trauma, COVID-19

## Abstract

**Background:**

Asian American (AA) community leaders, Native Hawaiian/Pacific Islander (NH/PI) community leaders, and allies in the United States Pacific Northwest expressed concern that there are families and children from AA communities and NH/PI communities who experience and witness acts of xenophobia and racism. This can cause racial trauma. The long-time practice of aggregating AA and NH/PI data contributes to erasure and makes it challenging to advance health equity, such as allocating resources. According to AAPI Data’s long-awaited report in June 2022, there are over 24 million AAs and 1.6 million NHs/PIs in the United States, growing by 40% and 30%, respectively, between 2010 and 2020. Philanthropic investments have not kept up with this substantive increase. The National Academies of Sciences, Engineering, and Medicine emphasized the need for effective partnerships to advance the health and well-being of individuals and communities in antiracism and system-level research.

**Objective:**

The aim of this community-based participatory research qualitative description study was to identify perceptions and experiences regarding racial discrimination, race-based stress, and racial trauma; intergenerational healing and resiliency; and sharing the body with science from key informants of an academic and community partnership to inform antiracism coalition work. This partnership includes academic researchers and community leaders from community-based organizations and a health care organization serving immigrant and marginalized communities, including AAs and NHs/PIs in the United States Pacific Northwest.

**Methods:**

In total, 10 key informants joined 1 of 2 participatory group discussions via videoconference for 2 hours in 2022. We used a semistructured and open-ended group interview guide. A qualitative participatory group-level assessment was conducted with the key informants and transcribed. Interpretations and meanings of the main points and the main themes were reflected upon, clarified, and verified with the key informants in real time. The field note–based data transcripts were manually coded using conventional content analysis. Reflexivity was used.

**Results:**

There were 6 main themes: prejudice plus power in racism definition and working in solidarity to counter lateral oppression/false sense of security, microaggression as multilayers, “not assimilationist by nature” and responding differently to white superiority, intergenerational- and identity-related trauma, what is healing among People of Color and through a lens of resiliency and intergenerational connection and knowledge, and mistrust and fear in the research and health care systems surrounding intentions of the body.

**Conclusions:**

The themes highlight the importance of internal and intergenerational healing from racial trauma and the need for solidarity among communities of color to combat white supremacy and colonization. This work was foundational in an ongoing effort to dismantle racism and uplift the community voice through a cross-sector academic and community partnership to inform antiracism coalition work.

## Introduction

### Background

Asian American (AA) community leaders, Native Hawaiian/Pacific Islander (NH/PI) community leaders, and allies in Oregon and Washington States in the United States Pacific Northwest expressed concern that there are families and children from AA communities and NH/PI communities who experience and witness acts of xenophobia and racism. The power of science needs to focus on social issues and at a system level [1]. There is an ongoing need to mobilize, engage, and partner across sectors with health care organizations and community-based organizations in community organizing to inform antiracism coalition work. The National Academies of Sciences, Engineering, and Medicine emphasized the need for effective partnerships between health care organizations and community-based organizations to advance the health and well-being of individuals and communities [1]. Cross-sector partnership collaboration is important, and there must be recognition and navigation consideration of the contextual differences in power dynamics between sectors, such as funding resources and capacity of workforces [1,2], while collaborating on determining priority issues and actionable steps. Authentic intentionality is essential in being fully inclusive in the use of language and in-depth conversations on the diversity of perspectives and experiences and be driven by community and grassroots organizations regarding said inclusive language [1]. Research evidence points to the importance of relationship building within a culturally safe context for long-term sustainability between community and academic partners as meaningful engagement, especially working with immigrants and marginalized communities, including AA communities and NH/PI communities that have experienced historical trauma, including diaspora [3-13]. Earning and sustaining trust through mobilizing, engaging, partnering, and discussing difficult areas, priority issues, and actionable steps with community-based and health care organizations serving immigrants and marginalized communities, including AA communities and NH/PI communities, in research and honoring cultures are vitally important. Sustainable authentic relationships to inform antiracism coalition work in support of effective prevention and health programs are crucial.

### Contextual Considerations in Performing Antiracism Work: Erasure, Impacts of Racism, Race-Based Stress, Racial Trauma, and Protective Factors

There are multiple contexts to consider and recognize in performing antiracism work and a pathway forward for a post–COVID-19 pandemic time.

#### Erasure

Although AAs and NHs/PIs are diverse racial-ethnic populations, these populations are often aggregated as a monolithic group in health and human services data. The negative impact of the long-time practice of aggregating AAs and NHs/PIs must be recognized—*erasure of communities*. This long-time aggregation practice contributes to erasure and makes it challenging to advance health equity. Although data are used in decision-making on priorities regarding allocating resources, for example, during public health emergencies, and informing public health officials and policies aimed at reducing health inequities [14,15], there has been minimal policy attention on the burden of COVID-19 among AAs and NHs/PIs [16].

AAPI Data is a nationally recognized publisher of demographic data and policy research on AAs and PIs [17]. In June 2022 of the second year of the COVID-19 pandemic, AAPI Data reported on the long-awaited disaggregated data for AAs and NHs/PIs [17]. AAs and NHs/PIs are among the fastest-growing population groups in the United States based on the 2020 Census data [17]. There are over 24 million AAs and 1.6 million NHs/PIs in the United States, and the AA and NH/PI populations grew by about 40% and 30%, respectively, between the 2010 and the 2020 Census [17]. Over 1.7 million AAs are undocumented immigrants [17]. AAPI Data asserted there is economic devastation of the COVID-19 pandemic among AAs and NHs/PIs, including individuals, families, communities, and nonprofit community-based organizations [17]. According to AAPI Data, philanthropic investments have not kept up with this substantial increase in demand [18].

#### Impacts of Racism

Although racism is defined in the literature in different ways that illustrate, in part, mechanisms (see Multimedia Appendix 1 [1,19-22]), an underpinning is that racism affects health and wellness [23,24] and is entrenched in culture [23,25]. According to the National Academy of Medicine, there is a need to have intention in the use of language regarding racism that is inclusive of Black, Indigenous, and People of Color, including AAs and NHs/PIs, while acknowledging and doing work toward or in addressing structural racism and unequal allocation of power and resources as root causes of health inequities [26]. Racism can include prejudice, discrimination, or antagonism toward a race or ethnic group [25] and bias [22]. Discrimination is the most studied aspect of racism [23].

#### Race-Based Stress

Prior to the COVID-19 pandemic, AAs’ experiences of racial discrimination were rarely brought to public attention due, in part, to the model minority myth that erroneously posits that AAs do not experience the negative consequences of racism in this country as evidenced by their upward mobility and educational attainment [27]. This phenomenon, wherein the higher socioeconomic status some AAs have is used to deny or erase their experiences of racism, is a specific feature of anti-Asian racism, but in general, Black, Indigenous, and People of Color face significant race-based stressors due to the institutional, systemic, and interpersonal racism present in White-dominant US society [28,29]. Race-based stressors are threats of harm or injury, humiliating or shaming events, and witnessing harm to People of Color [30,31]. These race-based experiences have short-term and long-term impacts on mental health and physical health among socially disadvantaged racial and ethnic populations, including among children as adverse childhood experiences that can negatively impact well into adulthood as chronic health conditions [23,31-35]. Although the field has focused primarily on the experiences and consequences of race-based stress among African American people [36], AAs and NHs/PIs, along with minoritized groups, also suffer significantly from race-based stress [31,37].

#### Racial Trauma

During the COVID-19 pandemic, the escalating anti-AA hate and anti–NH/PI hate incidents were brought to public attention: 10,905 incidents between March 2020 and December 2021 in the United States, of which 4632 (42.48%) occurred in 2020 and 6273 (57.52%) occurred in 2021 [38]. Between January 2020 and March 2020, evidence of anti-AA sentiments was prevalent internationally on Twitter as well [39]. These direct and indirect racialized attacks have impacted mental and physical health [40-42]. Xenophobia is the fear of strangers—someone who is different from self or dislike of or prejudice against people from other countries [43,44]. Experiencing or witnessing acts of xenophobia and racism can cause racial trauma [31,37]. Similar to posttraumatic stress disorder, racial trauma involves injuries. Racial trauma is real or perceived experiences of discrimination in danger-related events [37]. Racial trauma differs from posttraumatic stress disorder in that there are ongoing injuries where there is exposure and re-exposure to race-based stress [37]. “Cumulative racial trauma can leave scars for those who are dehumanized” [37]. Scars can be psychological wounds or physiological effects or both [37]. Prolonged psychological and physiological stress resulting from discrimination and antagonistic interactions can result in health consequences due to dysregulation or chronic hypercortisolemia, such as suppressing immunity and increasing the risk of chronic diseases [45].

#### Protective Factors

The following are examples of protective factors. Cultural humility and inclusion are essential to address the increased xenophobia and racial trauma that has intensified as a result of the COVID-19 pandemic [40]. The National Academies of Sciences, Engineering, and Medicine underscored research evidence that the feeling of belonging is also essential [22]. Researchers found higher levels of social support, including caregiver emotional and instrumental support and peer support, decrease the negative effect of discrimination on allostatic load that is the cumulative stress—*wear and tear*—the body experiences [32,46]. Racism is an everyday risk factor [47]. Movement from a deficit lens to an asset-based one is crucial in antiracism work regarding healing.

### An Organized Academic and Community Partnership to Inform Antiracism Coalition Work

An academic and community partnership was created among academic researchers and community leaders from the public Washington State University College of Nursing; the College of Agricultural, Human, and Natural Resource Sciences; and the School of Biological Sciences; the nonprofit Immigrant & Refugee Community Organization (IRCO) and its Pacific Islander & Asian Family Center (PI&AFC); the nonprofit Pacific Islander Community Association of Washington (PICA-WA); and PeaceHealth not-for-profit health care system in the United States Pacific Northwest. IRCO unites newcomers and long-time community members from around the world and serves the holistic needs of immigrants, refugees, and mainstream community members in Oregon, with reach to Southwest Washington [48]. IRCO provides culturally and linguistically specific social services, including health, to build new lives and become self-sufficient [48]. PICA-WA serves as a cultural home, centers community power, and advocates to further the wellness of Pacific Islander communities in Washington [49]. PeaceHealth serves Oregon, Washington, and Alaska and promotes healing through personal and community health, relieving pain and suffering, and treating each person in a loving and caring way [50]. Culturally responsive community-based participatory research has been shown to center community voices and actionable leadership for changes [3,4,10,11,29].

We are mindful that prior researchers have reported ethical issues regarding collaboration, such as needing additional time, financial issues, the extent of comfort to discomfort regarding sharing power, and disempowerment among ethnic minoritized groups [3]. We have a foundational diverse cross-sector partnership to inform antiracism coalition work.

### This Study

Cohen et al’s [51] developing an effective coalition guided this study, and they describe effective coalition building can achieve more widespread reach within communities, sharing information, providing a range of perspectives, and accomplishing more together. The antiracism coalition includes AAs, NHs/PIs, and allies collaborating alongside. Due to the substantive breadth of community needs in an antiracism context, conducting a community-based participatory research qualitative description study is necessary to explore the in-depth diversity of perspectives and experiences to inform antiracism coalition work. Thus, the aim of this community-based participatory research qualitative description study was to identify perceptions and experiences regarding racial discrimination, race-based stress, and racial trauma; intergenerational healing and resiliency; and sharing the body with science from the key informants of a cross-sector academic and community partnership to inform antiracism coalition work. This work was foundational on findings related to priority issues and actionable steps to inform antiracism coalition work for accountability, growth, and sustainability.

## Methods

### Study Design and Key Informant Participants

In this community-based participatory research, we used qualitative description. Findings are closer to the data, as given by participants, in qualitative description, as Sandelowski described it [52,53]. Qualitative description is still interpretative [53]. Qualitative description aligns well in this study and the use of the scientific qualitative participatory group-level assessment method (described later).

We describe the background of academic researchers and community leaders from the organizational partnership who are from the antiracism coalition because this contributed to in-depth participatory group discussions. There were academic multiple principal investigators (MPIs), of which one was an academic nurse PI and identified as Vietnamese American with a Guamanian Micronesian Islander background and a community-based participatory research background and another was an academic human development PI and identified as White with a psychology background. On behalf of the academic MPIs, the academic human development PI emailed key informant participants with written instructions and a secure password-protected Qualtrics online link to the combined research study consent and sociodemographic and background form and obtained electronic consent. This study was held virtually via videoconference during the COVID-19 pandemic. Key informants from an academic and community partnership of an antiracism coalition joined 1 of 2 participatory group discussions for about 2 hours in January or February 2022. The academic MPIs were facilitators, and an academic prevention science researcher with a Native American and Asian Indian background was a cofacilitator and recorded field note–based data transcripts in both participatory group discussions. We determined the participation size was sufficient to achieve codebook stability and for understanding the main themes with a scope of this study to inform antiracism coalition work, the high quality of data from participants, and the methodological study on data saturation by Hennink et al [54]. We spoke with community organizational leadership regarding a compensation amount based on a mutual understanding of seed funding availability. From a social justice and equity lens, we compensated to commensurate the needed time for each key informant community leader at US $50 per hour for 2 hours for a total of a US $100 Visa gift card at the end of a participatory group discussion.

### Ethical Considerations

This study was determined exempt by the Washington State University Human Research Protection Program (#19080-001).

### Concurrent Data Collection and Data Analysis: Qualitative Participatory Group-Level Assessment Method

We adapted the scientific qualitative participatory group-level assessment method [55]. The group-level assessment method is from a social justice lens, where there is active involvement of participants in generating data, analyzing data, and reflecting on interpretations and meanings, clarifying, and verifying in real time with academic researcher facilitators. Group-level assessment steps include climate setting, generating data, appreciating perspectives, reflecting, understanding the data, selecting themes, and taking action.

Vaughn and Lohmueller [55] developed the qualitative participatory group-level assessment method to provide timely and valid data. The academic nurse PI previously modified the group-level assessment method in innovative community-based participatory research work with AAs and Micronesian Islanders to be inclusive of a storytelling communication style [6,8]. We provided an overview of how we adapted the qualitative group-level assessment steps used in the participatory group discussions in this study (Multimedia Appendix 2).

We obtained a rich texture of perceptions and experiences with the qualitative group-level assessment method. The academic MPIs and cofacilitator engaged in discussions through reflection and clarification and verified the interpretations and meanings of the main points and identified the main themes with key informants in real time during the participatory group discussions to ensure trust in meaningful data interpretation [56]. Consensus was sought and achieved among key informants and academic researcher facilitators. We referred to the co-constructed grounding agreements for a collaborative space from the climate setting step (see Multimedia Appendix 2), and this helped in working through discrepancies and disagreements. For example, there was a difference in interpretation and meaning regarding “hypervisibilized” versus “hypervigilance,” and this was clarified and verified with participants. In another example, there were different understandings that were discussed through reflection, clarified, and verified on the intended interpretations and meanings regarding assimilation and white supremacy. We identified the main themes using nonspecific quantification, including direct and nuances in findings related to the purpose of the study. The academic MPIs and cofacilitator debriefed immediately after each participatory group discussion and reviewed the impressions of group processes and interactions that provided an additional depth of understanding. The academic MPIs provided participants with access to the field note–based data transcripts, and there was no mention of major concerns about discrepancies or disagreements. The academic MPIs discussed with key informants and mutually agreed for academic researchers do the lifting work in additional conventional content analysis, selecting example quotes for the identified main themes, naming the themes, and then sharing for review. The academic MPIs, cofacilitator, and 2 academic nurse researcher collaborators met 2 times for 1.5-2 hours to further examine the recorded field note–based data transcripts and reviewed the main points and identified the main themes generated with key informants during the participatory group discussions. Next, the field note–based data transcripts were manually coded using conventional content analysis and reviewed for example quotes that represented the identified main themes with supported original text [57]. Interpretation variances enhanced credibility. The academic MPIs provided participants with access to the coded field note–based data transcripts. The academic nurse PI read the transcripts and selected example quotes that seemed to be the most representative for the identified main themes, performed an initial round of naming the main themes, and then discussed with the academic human development PI. The initial names of the main themes were longer in length, and the academic nurse PI shortened the names of the main themes for clarity as a round. Next, the academic nurse PI shared the names of the main themes and example quotes with the academic human development PI, cofacilitator, and the participants. There was no mention of major concerns about discrepancies or disagreements. Reflexivity was used throughout the process as a technique to address the influence of personal biases on results.

## Results

### Background of Key Informants

There was a total of 10 key informants who work with immigrants and marginalized communities, including AAs and NHs/PIs. Of the 10 key informants, 5 (50%) are community leaders from the AA communities and NH/PI communities and 5 (50%) identify as allies collaborating alongside. The key informants were invited to report in their own terms. Of the 10 key informants, 2 (20%) were academic nurse researchers and identified as Black. Of the 8 (80%) key informant community leaders, 3 (38%) are immigrant and refugee community leaders, 1 (12%) is an immigrant and refugee community leader for Pacific Islanders, 2 (25%) are NH/PI community leaders, and 2 (25%) are health caregiver leaders. The 8 (80%) key informant community leaders reported race and ethnicities as follows: Pacific Islander; Black/Hispanic; Finnish; White Hispanic; Vietnamese and Mexican; Polynesian/Tongan; Southeast Asian and Bhutanese-Nepali; and Samoan, Korean/Chinese, and White. All key informants spoke English. In addition, Samoan, Spanish, Finnish, and Heritage/elementary Vietnamese were also spoken. Additional sociodemographic information is shown in Table 1.

**Table 1:** Sociodemographics of key informant community leaders (N=8).

Sociodemographic variables		Participants^a^
**Race and ethnicity background, n (%)**		
	Pacific Islander	1 (13)
	Polynesian/Tonga	1 (13)
	Samoan, Korean/Chinese, and White	1 (13)
	Southeast Asian and Bhutanese-Nepali	1 (13)
	Vietnamese and Mexican	1 (13)
	Black/Hispanic	1 (13)
	White Hispanic	1 (13)
	Finnish	1 (13)
**Immigrant status, n (%)**		
	Immigrated to the United States	4 (50)
	Born in the United States	3 (38)
	Unsure of status	1 (13)
Lived in United States (years), mean (SD)		29 (14)
**Gender, n (%)**		
	Female	5 (63)
	Male	2 (25)
	Nonbinary	1 (13)
Age (years), mean (SD)		39 (13)
**Education level, n (%)**		
	Undergraduate degree	5 (38)
	Graduate degree	3 (38)
**Type of work, n (%)**		
	Community and social service	6 (75)
	Management	1 (13)
	Health care practice	1 (13)
**Organizational leadership, n (%)**		
	NH/PI^b^ community leaders	2 (25)
	Immigrant and refugee community leader for Pacific Islanders	1 (13)
	Immigrant and refugee community leaders	3 (38)
	Health caregiver leaders	2 (25)
**State of residence, n (%)**		
	Washington	5 (63)
	Oregon	3 (38)

^a^The sum of percentages could be more than 100 because of rounding.

^b^NH/PI: Native Hawaiian/Pacific Islander.

### Themes

There were 6 identified main themes across participatory groups, main points, and example quotes. There were 3 identified main themes in participatory group 1: (1) prejudice plus power in racism definition and working in solidarity to counter lateral oppression/false sense of security, (2) microaggression as multilayers, and (3) “not assimilationist by nature” and responding differently to white superiority. There were 3 identified main themes in participatory group 2: (1) intergenerational- and identity-related trauma, (2) what is healing among People of Color and through a lens of resiliency and intergenerational connection and knowledge, and (3) mistrust and fear in the research and health care systems surrounding intentions of the body. We provided descriptive interpretations for each theme. The diversity of perspectives and experiences in this cross-sector organizing movement is crucial in naming together what is happening and collectively as an antiracism coalition set forth priority issues and actionable steps.

#### Participatory Group 1

##### Prejudice Plus Power in Racism Definition and Working in Solidarity to Counter Lateral Oppression/False Sense of Security

There were some participants who referred to “your definition of racism” as coming from the lens of an institutional definition rather than from that of what must be recognized in a racism definition. A racism definition needs to include power, in addition to prejudice thoughts, in the context of who has the power to make decisions of impact and whether they can recognize the extent of the impact of those decisions on People of Color. This racism working definition makes prejudice plus power explicit. An NH/PI community leader commented on prejudice plus power and how that upholds individual and structural racism:

…there should be a focus on individual prejudice—and need to clarify with the People’s Institute is prejudice plus power and have to mention the addition of power…have to be clear that racism is set up with how this country is made and the way it is run.

NH/PI community leader J, Pacific Islander background

Most participants talked about the issue of lateral oppression between communities of color and must work in solidarity against white supremacy and how lateral oppression and internalized oppression show up. Working in solidarity is the coming together to unite, including community leaders and members, such as mobilizing and community organizing within and across diverse communities of color. The positionality of the lens through which racism is viewed and from which context are necessary to clearly identify and name racism. An immigrant and refugee community leader for Pacific Islanders described lateral oppression through a territorialism lens as an internal conflict:

...lateral oppression between communities of color…viewed as racism, but I see it as territorial, as it is less and to be categorized in that form…to address that internal conflict and territorial impact of People of Color—prejudice within or between.

Immigrant and refugee community leader for Pacific Islanders, K, Polynesian/Tongan background

Two NH/PI community leaders described white superiority/oppression of People of Color where lateral oppression is a manifestation of internalized oppression. Proximity to whiteness is colorism and is a false sense of security. Most participants expressed concern about tension between different communities of color, and this is a challenge in coming together. Working alongside as allies is a movement forward. Internalized oppression can show up through imposter syndrome. Not being able to view self as leaders is an example of internalized oppression.

When we clearly identify racism…we have to be committed that we are working in solidarity with each other against white supremacy. We have to get away from the lateral oppression but using the term “racist” to describe tension between Black and Brown communities [referring to how the term “racist” is being used], we have to understand racism within the context of white supremacy and how to dismantle it…dynamic as internalized racial oppression—white superiority and minority inferiority—the mentality of People of Color of anti-Black sentiments or at least I’m not them mentality; this is a false sense of security. [referring to People of Color] are…targets of white supremacy…do not want to be the target.

NH/PI community leader J, Pacific Islander background

What came up for me is that internalized oppression shows up through imposter syndrome [how there are community members who are leaders within but not viewing self as leaders] and seems to be through manifestation.

NH/PI community leader K, Samoan, Korean/Chinese, and White background

##### Microaggression as Multilayers

Some participants talked about how coloring would be perceived by people within the same community to be more accepted. A Black academic nurse researcher described colorism within cultures and internal community as a lived experience example. There is an emphasis on complexion or perfect skin color.

My family is from Central America—I am told that I am not Black enough in America…colorism really does play in within cultures…light skin is in or light skin is out…how it reflects on myself and others—when the eyes are on me and when they are not on me…much emphasis on complexion or perfect skin color to be more accepted plays into that internal community and how those different complexions are feeling and experiencing different things.

Academic nurse researcher N, Black background

In another example, a Black academic nurse researcher described that taking care of children who are struggling and feeling inferior due to the color of their skin is constant despite being successful in academia. The parenting lifting support is ongoing.

Our kids have to deal with this too [referring to colorism]. Have a daughter who is a senior in college and see her struggle in high school and college and now just with the color of her skin and feeling inferior. She is winning awards and excelling academically, but she could never believe that she was that or feel that she is anything…As adults we try to deal, but as kids it is disheartening. I have to reassure her don’t worry about the color of your skin; you are intelligent, you are beautiful. They have to believe that the color of their skin doesn’t matter…they can accomplish so much—constantly we have to remind them [referring to her children] that they can accomplish much. After a while I get numb—maybe I’m just dealing with it.

Academic nurse researcher D, Black background

A health caregiver leader commented on the feeling of not belonging due to the color of their skin and what have seen in their home country and in the United States in taking care of clients:

[Referring to home country] Strata based on money and…neighborhood and what kind of house you have in Columbia…just your color and stop you from joining a group or being a part of a different group of people even if you have the economic means, you can still feel that you do not belong, and I see this in my practice in America [United States].

Health caregiver leader A, White Hispanic background

An immigrant and refugee community leader for Pacific Islanders described interaction experiences about how “look like belong where” or “do not belong where” were based on physical appearance and assumptions despite being capable, having an education, or having a high income. The perceived appearance of being a perpetual foreigner is ongoing and how those perceptions inform decisions and lifting efforts to belong.

Talking about education and economics for Pacific Islander needs…to make up credit, but the school automatically throws in a PE [referring to physical education]–related class or course even when the child is capable and bright. When I walk into a building, I intentionally turn to a white employee so that they can see my badge, and it is my responsibility to show them that I belong in this building. [I] was told there’s this big Mexican seen in the building. Even if you climb up the ladder income-wise or education-wise, there are still barriers and they’ll [referring to White people] always question you—like, how did you get there? Or they ask so “what position did you play?”, assuming that I had a football scholarship—and I am not athletic...I am referring [they]…White people or around a White audience; this is how they respond to me, that has been the experience.

Immigrant and refugee community leader for Pacific Islanders, K, Polynesian/Tongan background

##### “Not Assimilationist by Nature” and Responding Differently to White Superiority

An NH/PI community leader voiced that Indigenous People have not consented to the ways of governance, and the dignity of Pacific Peoples must be maintained in the process and what colonialism white supremacy at work means:

We are not assimilationists by nature. We are Indigenous People, and we have never consented to these ways of governance…We are not going to go along with the system aside from all the prizes; this idea that we can get rich by stepping on your neighbor—this endless consumerist wealth-holding culture…not going to be a culture that we can continue to be a part of. Being human that we should assimilate by nature is not true…The dignity of my peoples, of Pacific Peoples is maintained in this process, and in some ways how do we continue to foster this sense of sovereignty and strength that can only be actualized in community. Others we are all going to be killed off. Can be genetically PI [referring to Pacific Islander], but if you don’t have your culture, language, way of being then they are not PI, then white colonialism white supremacy worked. Live out human values and not assimilate to another people’s values.

NH/PI community leader J, Pacific Islander background

Participants discussed the use of a social justice lens. A health caregiver leader described being surrounded by white supremacy and how members are responding differently to the white supremacy culture and to different radicalized trauma, and the importance of supporting social justice warriors:

…[referring to Asian Americans and Native Hawaiians/Pacific Islanders] members are responding differently to the white supremacy culture and different to radicalized trauma…how community members are responding differently that we all swim in it—surrounding white supremacy…we have an opportunity to explore this further…this has been studied within the context of Black and White and not many studies that are looking into white supremacy culture among the AA, PI/NH communities, so this is a huge contribution and helps us [referring to health care system] to understand better how we can be of service of those who are suffering and how we can support and apply those social justice warriors.

Health caregiver leader R, Finnish background

#### Participatory Group 2

##### Intergenerational- and Identity-Related Trauma

All participants talked about how impactful discrimination and trauma are and how this causes intergenerational trauma, where their own cultural way of living and being that may not be perceived or experienced as protective is not passed down from generation to generation. An immigrant and refugee community leader stated examples:

The impacts of discrimination and trauma lead to the shed of identities and pushing away from their [referring in general to working with immigrant and refugee clients] cultures and causing intergenerational trauma. [Examples of losses] Community connections, language, food sovereignty, ways of knowing, spirituality.

Immigrant and refugee community leader B, Vietnamese and Mexican background

Participants discussed experiences and expressed not feeling like belonging and that connection with immigrant and refugee clients is needed. An immigrant and refugee community leader described a personal experience of having faced racism at a young age but not having the term at that time to name it as racism. Walking alongside clients in their journey is crucial so that they know what it is they are experiencing and that they are not experiencing this alone.

For me…identify with experience. I grew up in a refugee camp, and I never felt that I belonged there. It was clear at a young age that my identity, and how I face racism, but I didn’t have the definition of what it is. I knew that I was not treated equally…I didn’t have the “right” terms to describe what I was experiencing. This leads to the need to talk about experiences, how and why? Focusing on empowerment and liberation—navigating new systems and cultures and naming racism, I connected the dots as I learned more about American [US] society and culture. When working with people [referring to clients in general] in similar situations, I encourage them to see, feel, and speak to their experiences. Reflecting on experiences from a bigger context of society.

Immigrant and refugee community leader N, Southeast Asian and Bhutanese-Nepali background

Participants talked about in their working with immigrants and refugees that there are many people who do not have the words or full understanding to name what might be happening to them, and this can be an added stressor. An immigrant and refugee community leader voiced examples of internalization and hypervisibilization due to physical appearance and identities perceived/shaped by others and being pushed away from or out of the community:

…these interactions accumulate and create a barrier causing internalization where people [referring to working with immigrant and refugee clients] continue to feel pushed away. Internalizations—that all these interactions may be a trend due to how you look leading to feeling pushed away from community. Terminology or full concepts may not be fully understood via trends—an incident happens, and they only have their own assumptions as to why the event might be happening.

Immigrant and refugee community leader B, Vietnamese and Mexican background

It can create this internalization of feeling that it is about them [referring to working with immigrant and refugee clients] or what might be common stereotypes and multifacets of their identity. When there are no words or understandings of the full concept, then sometimes it can translate to some incident, and they only have their own assumptions as to why something is happening. This causes an additional stressor, feeling pushed out of community or where they become common and hypervisibilized. Hypervisibilization to me is realizing that people are overattentive to you for whatever reason, or the reverse. A similar response when you think—oh I need to watch out for this, so I don’t experience this again.

Immigrant and refugee community leader B, Vietnamese and Mexican background

In another example, an immigrant and refugee community leader voiced an inclusion concern—intersectional identities and further trauma experiences following immigration with fear of not being accepted:

…racial discrimination…the trauma that occurs after immigration and integration makes me think about how queer, and other folks with intersectional identities, are further discriminated against, even intercommunity, because of fear around further trauma from not being accepted on individual, structural, and institutional basis.

Immigrant and refugee community leader K, Black/Hispanic background

##### What Is Healing Among People of Color and Through a Lens of Resiliency and Intergenerational Connection and Knowledge

Participants talked about healing and resiliency together as healing from trauma, although participants raised the question about how truly one can heal since trauma keeps occurring. An immigrant and refugee community leader voiced the strength and fluidity of intergenerational connection and knowledge exchange as a form of healing, how holding the trauma in or deciding not to share it with other people is a concern, trying to deeply understand the trauma or not want to be judged by other people, and needing to name what is happening and the impacts before one can truly heal.

The resilience part is coming out…talking about mental wellness training and the content… thinking of trauma as an injury. I see healing and resiliency as a healing for trauma, since the wounds are still opened and injured repeatedly…I focus on resiliency as they [referring to immigrant and refugee clients in general] turn inward with social cohesion and finding support and within communities. Broader understanding of certain phrases or terminology, as entities do not want to name it as what it is. Notion that racism and systemic oppression is historical or in the past, although it is happening today and in what ways. Gentrification and the impacts of this on communities of color—how can we truly heal if we do not learn the impacts, healing can’t happen when you don’t know why you’re impacted or what you are trying to heal from.

Immigrant and refugee community leader K, Black/Hispanic background

Intergenerational connection has been a method community [referring in general to what is known in working with immigrants and refugees] has used to come together to both learn and come to terms with their history. Even if this does not include learning from an elder, but just spending time with them and being okay with what comes and doesn't come out of it. People don’t talk about their traumas. There are harmful forms of “integration” and “assimilation” (shedding language, identity, culture) that can leave people to struggle in accepting themselves and their stories, which does not allow for healing/resilience.

Immigrant and refugee community leader K, Black/Hispanic; background

Participants raised questions about what is healing and in what ways People of Color heal. This can have emotional and mental health impacts, where being resilient is already a must. Another immigrant and refugee community leader commented on experiences working in general with People of Color who do not know where to go for rest and play:

…the community [referring in general to People of Color] is often trying to continue to be resilient and there is guilt when one seeks means of rest. And spaces of rest/play can lack [minimal] representation…so community is unsure where to go for rest/play options.

Immigrant and refugee community leader B, Vietnamese and Mexican background

##### Mistrust and Fear in the Research and Health Care Systems Surrounding Intentions Regarding the Body

Participants talked about fear and concern surrounding the body and not knowing what exactly the intentions for science are. Mistrust and trauma from immigration and refugee experience must be recognized. An immigrant and refugee community leader raised questions that could be stemming from mistrust and trauma from immigration or refugee camp experience:

Fear and concern surrounding the body and not knowing what intentions are and the mistrust of institutions and science, this idea of who is it and who is behind science? What are they doing with my information and my body? It is intimidating not knowing what is being asked of you and the next steps. Although we do care about future generations and our health, but initially there is fear…there's mistrust and trauma from refugees and immigrants around incidents that might have occurred to them/their family when immigrating/in refugee camps.

Immigrant and refugee community leader B, Vietnamese and Mexican background

Mistrust in the United States regarding treatment of People of Color in medical institutions is part of past and current history. Another immigrant and refugee community leader described this from the context of whether there is authentic intention in supporting communities and healing or the concern whether is just a datapoint—seeing the humanity in scientific work and meaning for healing in communities.

We cannot ignore the mistrust in the America [referring to United States] context how medical institutions have treated People of Color and how their bodies have been used, as well as not having access to health care. Medical institutions have used People of Color as commodities. What does access and the process look like?...I’ve been through experiences with family or my mother, and the doctors think that she knows the terminology or know what medications she needs to take and what they all entail…become a datapoint instead of their [referring in general to working with immigrant and refugee clients] own experiences and their feelings…is it about supporting communities and healing or is it just a datapoint?

Immigrant and refugee community leader N, Southeast Asian and Bhutanese-Nepali background

Participants talked about mistrust in research and health care systems in the context of positions of power. An immigrant and refugee community leader voiced about the power gap and dynamic in research and health care and recommendations to view as a social contract between providers, researchers, and scientists and patients/communities of color/People of Color to gain trust and to use a historical trauma lens:

…There is a valid reason why communities of color do not trust the system. The researchers are providers have to build up the trust and how can we lessen the gap of that power dynamic? [Examples] Providers, researchers, and scientists have power/knowledge versus patients/communities of color/People of Color, in relationship with each other. What should the social contract be between these two? Those in power need to come to enter relationships and gain trust, acknowledged the historical trauma done to People of Color and interact with this lens…

Immigrant and refugee community leader K, Black/Hispanic background

## Discussion

### Principal Findings

In this community-based participatory research qualitative description study, we identified 6 main themes: prejudice plus power in racism definition and working in solidarity to counter lateral oppression/false sense of security; microaggression as multilayers; “not assimilationist by nature” and responding differently to white superiority; intergenerational- and identity-related trauma; what is healing among People of Color and through a lens of resiliency and intergenerational connection and knowledge; and mistrust and fear in the research and health care systems surrounding intentions of the body.

Participants highlighted the importance of solidarity among communities of color and the need to recognize and combat lateral oppression. Lateral oppression or violence is rooted, in part, in the deliberate efforts of the settler-colonial project to erase the existence of Indigenous Peoples. The oppressive regulation of access to resources and land, blood quantum laws, and US hegemony feed the competition and aggression within Indigenous communities [58]. Participants’ assertion of the need to recognize and combat internalized racial oppression and the ways it manifests as lateral violence or oppression echoes calls to action from scholars who see the minimal awareness of and empirical attention to these phenomena as critical barriers to antiracist progress [59]. A refusal to engage in lateral oppression connects to the theme of “not assimilationist by nature” and the rejection of settler-colonialism by Indigenous Pacific Peoples. Participants also shared concerns regarding cultural assets that are lost to assimilation and the resilience and strengths inherent in traditional practices and knowledge sharing, including intergenerational relationships. Resistance to assimilation aligns with efforts to decolonize AAs’ and NHs’/PIs’ understandings of self and community. One recent study fostered resilience by using online forums as safe(r) spaces for collective identity work among AAs and NHs/PIs [60]. The virtual videoconference-based participatory groups in our study were similar, in that they served as spaces for community members to process and explore collective pathways forward for coalition building and decolonization.

Understanding the ongoing impacts of colonization for Indigenous Peoples also grounds the theme of intergenerational trauma in the concept of historical trauma or the “cumulative emotional and psychological wounding across generations including one’s own lifespan” [61]. The role of historical trauma in the healthy inequities experienced by American Indians and Alaska Natives is well documented [62]. Comparable awareness is needed of the impact of historical trauma among Indigenous Pacific Peoples as well. Antiracism coalition work must involve an appreciation for the oppression perpetrated and perpetuated by settler-colonialism and a commitment to decolonization. As described by scholars of decolonization, this work must involve true and authentic honoring of Indigenous sovereignty and cannot be reduced to symbolic gestures that ultimately uphold white supremacist structures [63].

The stories shared by participants also communicated the far-reaching impacts of white supremacy and white superiority in people’s lives and communities, including through experiences of microaggressions. Sue et al [64] defined microaggressions as “brief and commonplace” racialized attacks that can include microassault, microinsult, and microinvalidation. Some evidence suggests that AAs experience microaggression more than other marginalized and minoritized groups [65]. For AAs, microaggressions include the racist trope of the “perpetual foreigner”—the treatment of AAs as though they do not belong in their own country. This treatment has been linked to lower levels of social belonging and life satisfaction [66]. The long-standing practices of erasure of AAs and NHs/PIs in health data and the dismissal or downplaying of experiences of racism in these communities can be viewed through the lens of microaggression as well. Along with addressing larger systems of oppression, antiracism work must address the daily, perhaps unintentional, racialized acts that perpetuate harm and undermine the well-being of AAs and NHs/PIs. For instance, People of Color and White allies who witness racial microaggressions can perform microinterventions that acknowledge and disarm the microaggressions and validate the experiences of the targets of those attacks [67].

The second participatory group discussed topics related to mistrust and fear among their families and community members when interfacing with health care systems. The absence of or minimal cultural understanding and sensitivity results in racialized discomfort that can discourage community members from seeking medical care or participating in health research [68]. The long practice of health data aggregation contributes to erasure of rich and distinct cultural communities and makes it challenging to advance health equity. Koholokula et al [14] reported that NH/PI communities have been calling for better data collection and analysis via disaggregation of NH/PI data apart from AA data and for accuracy in public health reports and data surveillance systems, even prior to the COVID-19 pandemic. The extent of reach and addressing the needs of communities are often limited by inadequate data disaggregation [69-72]. The perspectives shared by participants in this study inform the continuing work of our research team partnership, and this uplifts voices and center stories regarding racialized experiences in accessing health care among multigenerational families within AA communities and NH/PI communities. Collective conversations about sharing the body with science or medicine are also taking place with an understanding of parents and relatives as Family Leaders. This approach is consistent with both community-based participatory research and citizen science [73] to leverage community knowledge and leadership to address and dismantle racialized barriers to health and well-being.

### Limitations

This study has some limitations to consider. The qualitative study design prioritized uplifting participants’ unique voices, and the community-based participatory research approach involved deep engagement and relationship building with specific community partners. As such, the results of this work are not necessarily generalizable beyond the people involved or the communities served. The key informant community leaders drew on their experiences working with diverse groups of AAs and NHs/PIs. We recognize that their perspectives cannot represent all AAs and NHs/PIs, however, and we resist understanding these groups as monoliths with singular identities or experiences. There is still much research to be done to dismantle racism against and reverse the erasure of AAs and NHs/PIs.

### Conclusion

The identified themes from our community-based participatory research qualitative description study highlight the importance of internal and intergenerational healing from racial trauma and the need for solidarity among communities of color to combat white supremacy and colonization. This work was a foundational step in an ongoing effort to dismantle racism and uplift the community voice through a cross-sector academic and community partnership to inform antiracism coalition work.

## Appendix

Multimedia Appendix 1 Examples of racism definitions that illustrate, in part, mechanisms.

Multimedia Appendix 2 Qualitative participatory group-level assessment: overview of adapted steps. We provided an overview of how we adapted the qualitative participatory group-level assessment steps in the participatory group discussions in this community-based participatory research qualitative description study.

## Abbreviations

AA: Asian American
IRCO: Immigrant & Refugee Community Organization
MPI: multiple principal investigator
NH/PI: Native Hawaiian/Pacific Islander
PICA-WA: Pacific Islander Community Association of Washington
