# Supplementary material for: An Antiracism Community-Based Participatory Research With Organizations Serving Immigrant and Marginalized Communities, Including Asian Americans and Native Hawaiians/Pacific Islanders in the United States Pacific Northwest: Qualitative Description Study With Key Informants
Source: Asian Pac Isl Nurs J. 2023 Jan 11;7:e43150. doi: 10.2196/43150 (PMC9976990; doi:10.2196/43150)
Supplement: Multimedia Appendix 1 [file apinj_v7i1e43150_app1.pdf]

Multimedia Appendix 1: Examples of racism definitions that illustrate, in part, mechanisms.

Sources	Examples of racism definitions
**Implicit Bias**<br>National Academies of Sciences, Engineering, and Medicine the science of implicit bias workshop[a]	"Commonly defined as any unconscious or unacknowledged preferences that can affect a person's beliefs or behaviors, and in particular, an unconscious favoritism toward or prejudice against people of a certain race, gender, or group that influences one's own actions or perceptions."
**Racism**<br>National Academies of Sciences, Engineering, and Medicine addressing diversity, equity, inclusion, and anti-racism in 21st century workshop[b]	"The devaluation and the denial of rights, dignity, and value of individuals due to their race or geographical origin."
**Racism**<br>National Commission to Address Racism in Nursing[c]	"Assaults on the human spirit in the form of biases, prejudices, and an ideology of superiority that persistently cause moral suffering and perpetuate injustices and inequities."
**Racism**<br>Shaio and Woody[d]	From a sociological perspective, can be contextualized into three categories: attitudes, culture, and structure.<br><br>Attitudes assesses mental states that can affect and reflect an individual's behaviors, including deliberate inaction.<br><br>Culture refers to the schema used to make sense of self, the world around them, and the connotation of group membership.<br><br>Structure can be framed in two circumstances: one's immediate circumstances and the impact of social relations on how an individual makes sense of the broader world.
**Individual Racism**<br>The Aspen Institute[e]	Overt or covert that is subtle acts of hate, bias, prejudice, or discrimination toward another based on race.
**Structural Racism**<br>The Aspen Institute[e]	Policies, institutional practices, cultural, and social interactions that reinforces racial inequity. Historic, perpetual privileges of and preference of White bodies, and imbedded within the social, capitalist, political systems through which we all exist.

**Institutional Racism –** The Aspen Institute[e]	Policies, practices that benefit White people over Black, Indigenous, and People of Color individuals, often intentional or inadvertently bias is upheld.

[a]National Academies of Sciences, Engineering, and Medicine. The Science of Implicit Bias: Implications for Law and Policy. Proceedings of a Workshop–in Brief. Washington, DC: The National Academies Press ; 2021 [doi: 10.17226/26191]

[b]National Academies of Sciences, Engineering, and Medicine. Addressing Diversity, Equity, Inclusion, and Anti-Racism in 21st Century STEMM Organizations: Proceedings of a Workshop–in Brief. Washington, DC: The National Academies Press; 2021 [doi: 10.17226/26294]

[c]National Commission to Address Racism in Nursing. Defining Racism. URL: https://www.nursingworld.org/~4a0e54/globalassets/practiceandpolicy/workforce/commission-to-address-racism/final-defining-racism.pdf [accessed 2022-07-02]

[d]Shiao J, Woody A. The meaning of "racism". Sociol Perspect. 2021;64(4):495-517. doi:10.1177/0731121420964239

[e]The Aspen Institute. 11 Terms You Should Know to Better Understand Structural Racism. URL: https://www.aspeninstitute.org/blog-posts/structural-racism-definition/ [accessed 2022-02-01]
