# Supplementary material for: An Antiracism Community-Based Participatory Research With Organizations Serving Immigrant and Marginalized Communities, Including Asian Americans and Native Hawaiians/Pacific Islanders in the United States Pacific Northwest: Qualitative Description Study With Key Informants
Source: Asian Pac Isl Nurs J. 2023 Jan 11;7:e43150. doi: 10.2196/43150 (PMC9976990; doi:10.2196/43150)
Supplement: Multimedia Appendix 2 [file apinj_v7i1e43150_app2.pdf]

Multimedia Appendix 2: Qualitative participatory group-level assessment - overview of adapted steps.

We provided an overview on how we adapted the qualitative participatory group-level assessment steps in the participatory group discussions in the community-based participatory research qualitative description study.

Group-level assessment steps	Overview in participatory group discussions
**Climate setting step**	Built trust and rapport and co-created a comfortable atmosphere for engagement and discussion. We opened with a dinámica (i.e., social learning activity) and co-constructed grounding agreements for a collaborative space.<br><br>Opened with a dinámica (i.e., social learning activity):<br><br>An academic nurse researcher facilitated and invited participants to share their thoughts on the following.<br>• How would you like to be addressed in this study space for the purpose of interacting? For example, During our time together, I would like to go by _________. My pronoun(s) is/are _________.<br>• What is something that you like to do as a hobby or activity other than work related? This is in the context of caring. For example, I like to ______________________________. I welcome someone to step up to go next."<br><br>Co-constructed grounding agreements for a collaborative space:<br>• To honor diversity and disaggregate Asian Americans and Native Hawaiians/Pacific Islanders as diverse racial-ethnic groups<br>• To participate and stay engaged.<br>• Commit to a brave and safe space.<br>• Honor self and people in this space with care: Honoring being both knowers and learners.<br>• Deeply listen to understand.

	• Step up to share and step back so that others can share.
**Generating data step**	Engaged participants with broad prompts and questions that encouraged participants to direct their thoughts and experiences.<br><br>We used a semi-structured and open-ended participatory group discussion guide with prompts and follow-up questions and provided to participants via share screen feature in the videoconference.<br><br>• Prompts included, for example: *racism context – racial discrimination, race-based stress, and racial trauma; intergenerational healing and resiliency; sharing the body with science.*<br>• *Follow*-up questions included, for example: *Can you give us an example or examples when you said...* and *To tell us more about...*<br><br>In addition to the academic researcher co-facilitator having recorded field notes-based data transcripts in both participatory group discussions, participants were also encouraged and used the written chat feature in the videoconference as an option and incorporated into the field-notes based data transcripts.
**Appreciating perspective step**	Reviewed the main points and encouraged participants to share in the virtual space.
**Reflecting step**	Reflected with participants on the interpretations and meanings of perspectives and experiences, clarified, verified, and added summative thoughts.
**Understanding the data step**	Identified with participants potential common themes from the content as main themes.
**Selecting themes step**	Selected and prioritized main themes with participants.
**Action step**	Worked on determining priority issues and action planning and steps with participants.
